# Serum microRNAs as new biomarkers for detecting subclinical hemolysis in the nonacute phase of G6PD deficiency

**DOI:** 10.1038/s41598-024-67108-4

**Published:** 2024-07-11

**Authors:** Kanyarat Boonpeng, Tatsuki Shibuta, Yoshitaka Hirooka, Kasem Kulkeaw, Duangdao Palasuwan, Tsukuru Umemura

**Affiliations:** 1 Program in Clinical Hematology Sciences, Department of Clinical Microscopy, Faculty of Allied Health Sciences, Chulalongkorn University, Bangkok, 10330 Thailand; 2 Graduate School, Department of Medical Technology and Sciences, International University of Health and Welfare, 137-1 Enokizu, Okawa, 831-8501 Japan; 3 Siriraj Integrative Center for Neglected Parasitic Diseases, Department of Parasitology, Faculty of Medicine Siriraj Hospital, Mahidol University, 2, Wanglang Road, Bangkok Noi, Bangkok, 10700 Thailand; 4 Oxidation in Red Cell Disorders Research Unit, Department of Clinical Microscopy, Faculty of Allied Health Sciences, Chulalongkorn University, Bangkok, 10330 Thailand; 5 Clinical Laboratory, Kouhoukai Takagi Hospital, 141-11 Sakemi, Okawa, 831-0016 Japan

**Keywords:** Glucose-6-phosphate dehydrogenase, Enzymopathy, microRNAs, Hemolysis, Biomarkers, Medical research, Molecular medicine

## Abstract

Glucose-6-phosphate dehydrogenase (G6PD) deficiency is one of the most common enzymopathies worldwide. Patients with G6PD deficiency are usually asymptomatic throughout their life but can develop acute hemolysis after exposure to free radicals or certain medications. Several studies have shown that serum miRNAs can be used as prognostic biomarkers in various types of hemolytic anemias. However, the impact of G6PD deficiency on circulating miRNA profiles is largely unknown. The present study aimed to assess the use of serum miRNAs as biomarkers for detecting hemolysis in the nonacute phase of G6PD deficiency. Patients with severe or moderate G6PD *Viangchan* (871G > A) deficiency and normal G6PD patients were enrolled in the present study. The biochemical hemolysis indices were normal in the three groups, while the levels of serum miR-451a, miR-16, and miR-155 were significantly increased in patients with severe G6PD deficiency. In addition, 3D analysis of a set of three miRNAs (miR-451a, miR-16, and miR-155) was able to differentiate G6PD-deficient individuals from healthy individuals, suggesting that these three miRNAs may serve as potential biomarkers for patients in the nonhemolytic phase of G6PD deficiency. In conclusion, miRNAs can be utilized as additional biomarkers to detect hemolysis in the nonacute phase of G6PD deficiency.

## Introduction

Glucose-6-phosphate dehydrogenase (G6PD) deficiency is one of the most common enzymopathies, affecting 400 million people worldwide^1^. The estimated prevalence of G6PD deficiency in Thailand ranges from 3 to 18%^2^. The G6PD *Viangchan* form is the most common variant in Thailand, accounting for approximately 54% of the identified mutations^3^. Individuals with G6PD deficiency are usually asymptomatic^4^, but hemolysis can be commonly triggered by certain medications and infections^4,5^. When G6PD-deficient erythrocytes are exposed to severe hypoxia, free radicals, or some medications, such as primaquine, erythrocytes become highly vulnerable to hemolysis^4,5^. For patients with severe hemolysis, prompt transfusions can substantially and rapidly improve their clinical course^4^. In Thailand, G6PD deficiency is typically diagnosed only in symptomatic patients, namely, in patients experiencing acute hemolytic attacks. Patients with G6PD deficiency and oxidative stress also experience damage to other organs, called extraerythrocytic manifestations, endothelial cell damage, infection, and coagulation cascade activation^6–8^. However, there are limited useful biomarkers for assessing hemolytic status in asymptomatic patients.

During hemolysis, numerous components of erythrocytes, including microRNAs (miRNAs), are released into the blood or extracellular space^9^. Previous reports have shown that circulating miRNAs can be used as diagnostic or prognostic biomarkers for various hemolytic anemias^10–13^, specifically miR-451a and miR-16, which are abundant in erythrocytes^9,14^. The serum miR-451a concentration has been shown to be elevated in patients with severe beta-thalassemia/HbE illness^11,12^. Low levels of miR-510 and miR-629 have been observed in patients with severe sickle cell disease^10^. However, the impact of G6PD deficiency on miRNA profiles is largely unknown. The present study aimed to characterize the serum miRNAs in patients with G6PD deficiency. Since G6PD deficiency primarily impacts erythrocytes, six blood cell-derived miRNAs were selected based on previous in vitro^15–24^ and clinical studies^25^. Specifically, miR-451a, miR-16, miR-155, and miR-144 are involved in regulating erythropoiesis^18,22,24^, regulating oxidative stress^20,21,23^, and maintaining erythrocyte homeostasis and lifespan^16,19^. Blood cells serve as major sources of circulating miRNAs and potentially contribute to the release of intracellular miRNAs from blood cells into the bloodstream^25^. Consequently, additional blood cell-derived miRNAs, such as miR-223 from granulocytes^17^ and miR-126 from platelets^15^, were also investigated.

## Results

### Clinical data of G6PD-deficient subjects

The clinical data of the 11 control subjects, 5 subjects with moderate G6PD deficiency, and 8 subjects with severe G6PD deficiency are shown in Table 1. The following mean ages of the patients were not significantly different among the groups: 29.38 ± 11.25 years in the severe G6PD-deficient group, 26.40 ± 12.64 years in the moderate G6PD-deficient group, and 24.73 ± 3.72 years in the normal group (Table 1). Moreover, the erythrocyte indices did not significantly differ among the subjects (*P* < 0.05), and the erythrocytes in all subjects were normochromic and normocytic. Patients with severe G6PD deficiency had significantly decreased G6PD activity (1.11 ± 0.90) compared to normal controls (15.96 ± 3.03; *P* < 0.001), while the G6PD activity of those with moderate G6PD deficiency (8.63 ± 0.67) did not significantly differ compared to normal controls.

**Table 1:** Hematological parameters of the normal and G6PD-deficient subjects recruited for the study.

Parameters	G6PD normal (n = 11)	Moderate deficiency (n = 5)	Severe deficiency (n = 8)
Age	24.73 ± 3.72 (20–30)	26.40 ± 12.64 (20–49)	29.38 ± 11.25 (20–48)
Sex	Male = 4, Female = 7	Female = 5	Male = 7, Female = 1
Hemoglobin, g/dL	13.51 ± 1.60	12.38 ± 1.12	13.08 ± 1.33
Hematocrit, %	41.20 ± 4.45	38.24 ± 2.93	40.23 ± 3.12
RBC count, × 10^6^/µL	4.82 ± 0.52	4.51 ± 0.47	4.77 ± 0.48
MCV, fL	85.78 ± 6.74	85.34 ± 9.87	84.91 ± 8.78
MCH, pg	28.12 ± 2.66	27.64 ± 3.83	27.66 ± 3.64
MCHC, g/dL	32.73 ± 0.72	32.32 ± 1.03	32.48 ± 1.08
RDW, %	14.36 ± 1.51	14.18 ± 0.81	13.54 ± 0.93
WBC, × 10^3^/µL	6.89 ± 1.40	5.96 ± 1.62	6.45 ± 1.72
Neutrophil, %	60.82 ± 7.43	60.00 ± 10.86	56.38 ± 5.07
Lymphocyte, %	29.09 ± 5.39	30.00 ± 9.97	33.50 ± 4.78
Monocyte, %	6.36 ± 2.50	7.80 ± 1.30	6.88 ± 1.89
Eosinophil, %	3.82 ± 3.19	1.40 ± 0.55	2.63 ± 2.45
Basophil, %	0.82 ± 0.40	0.80 ± 0.45	0.75 ± 0.46
PLT count, × 10^5^/µL	2.91 ± 0.75	2.93 ± 0.52	2.66 ± 0.29
RBC Morphology	Normochromic Normocytic RBC	Normochromic Normocytic RBC	Normochromic Normocytic RBC
Quantitative G6PD activity, IU/gHb	15.96 ± 3.03	8.63 ± 0.67	1.11 ± 0.90*

*Statistically significant difference (*P* < 0.001).

Data are presented as the mean ± standard deviation (SD).

*RBC* red blood cell, *MCV* mean corpuscular volume, *MCH* mean corpuscular hemoglobin, *MCHC* mean corpuscular hemoglobin concentration, RDW red blood cell distribution width, *WBC* white blood cell, *PLT* platelet, *G6PD* glucose-6-phosphate dehydrogenase.

### Limitations of biochemical indices for detecting subclinical hemolysis

No differences in the serum levels of potassium (K +), aspartate transaminase (AST), and lactate dehydrogenase (LDH), which are used as biochemical markers of hemolysis, were found among the three groups. The free serum Hb levels did not differ among the three groups, with levels of 4.82 ± 2.20, 4.63 ± 1.18, and 4.84 ± 2.00 mg/dL for the normal (n = 11), moderate deficiency (n = 5), and severe deficiency (n = 8) groups, respectively. The average concentrations of the following parameters did not differ among the three groups: 4.34 ± 0.66 mEq/L potassium, 16.45 ± 3.24 U/L AST, and 154.27 ± 24.94 U/L LDH in the normal group; 4.00 ± 0.46 mEq/L potassium, 19.00 ± 6.75 U/L AST, and 138.60 ± 35.05 U/L LDH in the moderate deficiency group; and 4.48 ± 0.50 mEq/L potassium, 17.25 ± 2.24 U/L AST, and 145.13 ± 33.49 U/L LDH in the severe deficiency group (Table 2). These results indicated that hemolysis was undetectable with the NanoDrop method and biochemical testing in patients with G6PD deficiency.

**Table 2:** Serum hemolysis indices of the recruited subjects.

Hemolysis indices	Normal (n = 11)	Moderate (n = 5)	Severe (n = 8)	Reference range
Free Hb	4.82 ± 2.20	4.63 ± 1.18	4.84 ± 2.00	< 5 mg/dL
K +	4.34 ± 0.66	4.00 ± 0.46	4.48 ± 0.50	3.6–5.0 mEQ/L
AST	16.45 ± 3.24	19.00 ± 6.75	17.25 ± 2.24	10–40 U/L
LDH	154.27 ± 24.94	138.60 ± 35.05	145.13 ± 33.49	124–222 U/L

Data are presented as the mean ± standard deviation (SD).

*Hb* hemoglobin, K+, potassium, *AST* aspartate aminotransferase, *LDH* lactate dehydrogenase.

### Serum miRNA analyses

**Figure 1:**
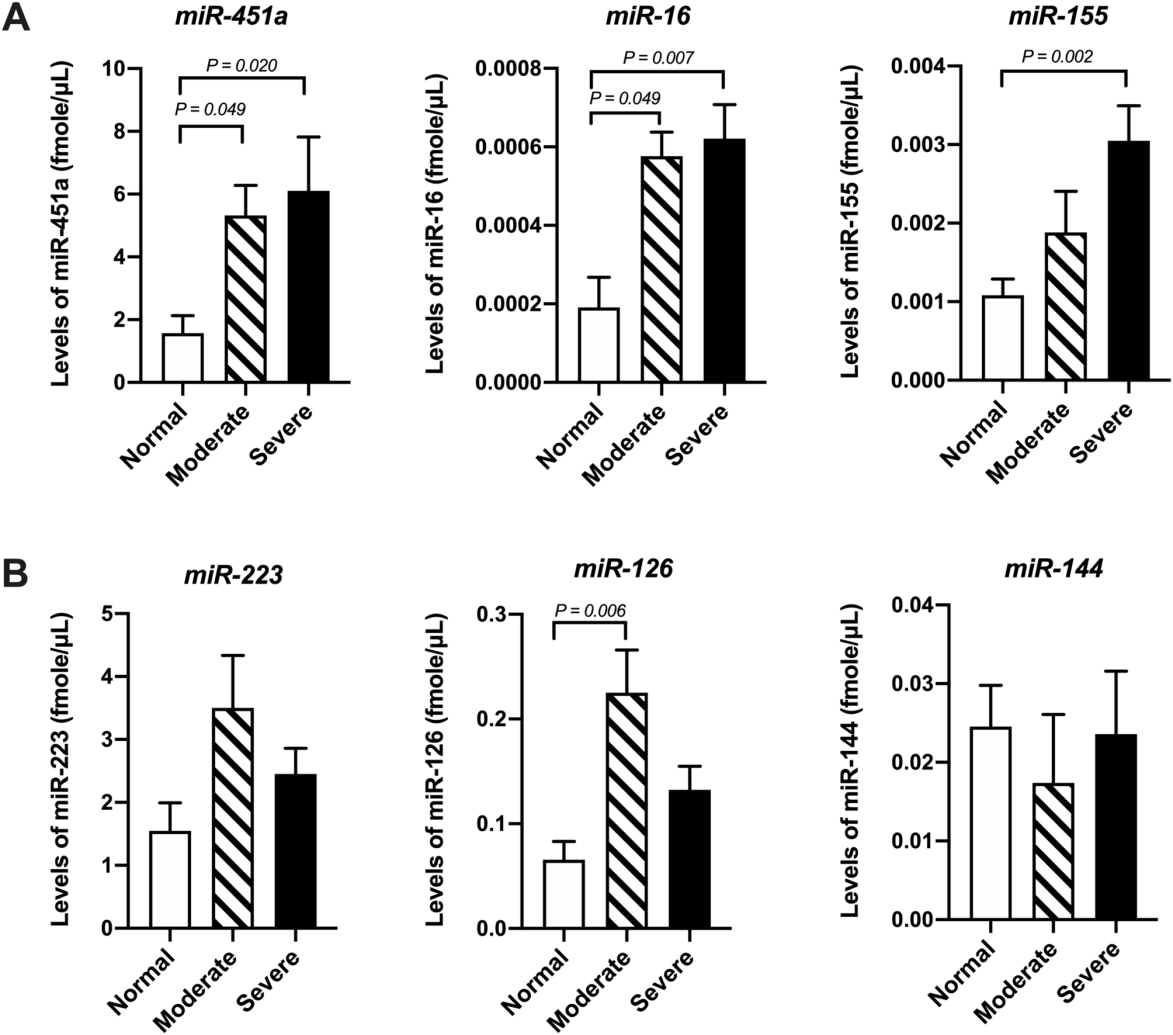
Increased levels of serum miR-451a, miR-16, and miR-155 in patients with G6PD deficiency. The ∆C_t_ values of the miRNAs were calculated using the C_t_ values of the internal control cel-miR-39. (**A**) Patients with G6PD deficiency had significantly higher serum miR-451a, miR-16, and miR-155 levels compared to the normal control (Ctrl) subjects, whereas (**B**) the levels of miR-223, miR-126, and miR-144 did not significantly differ. The statistical analyses were performed using the Kruskal‒Wallis test. A *P* value < 0.05 was considered to indicate statistical significance.

The level of miR-451a was significantly higher in patients with moderate (5.32 ± 0.95 fmol/µL, *P* = 0.049) or severe G6PD deficiency (6.11 ± 1.71 fmol/µL, *P* = 0.020) than in normal controls. Similarly, the levels of miR-16 were significantly higher in moderate cases (0.0005 ± 0.00006, *P* = 0.049) and severe cases (0.0006 ± 0.00008, *P* = 0.007) than in normal cases, and the level of miR-155 was significantly higher in the severe group (0.003 ± 0.0004, *P* = 0.002) than in the normal control group (Fig. 1A). These results showed that the serum levels of erythrocyte-related miRNAs, especially miR-451a, increased in patients with G6PD deficiency even during the nonacute hemolytic phase. The level of miR-223, which is expressed mainly in granulocytes, was not significantly increased in patients with moderate (3.503 ± 0.832) or severe G6PD deficiency (2.451 ± 0.407 fmol/µL). The level of platelet-related miR-126 significantly increased in moderate cases (0.225 ± 0.041 fmol/µL, *P* = 0.006) but not in severe cases (0.132 ± 0.022 fmol/µL). Similarly, the level of miR-144 was not significantly altered in moderate (0.017 ± 0.008 fmol/µL) or severe cases (0.024 ± 0.007 fmol/µL) (Fig. 1B). Additionally, the elevation of these serum miRNAs was not influenced by confounding factors, such as the age of the recruited subjects (Supplementary Fig. 1).

### Correlation between miRNA levels and G6PD activity

**Figure 2:**
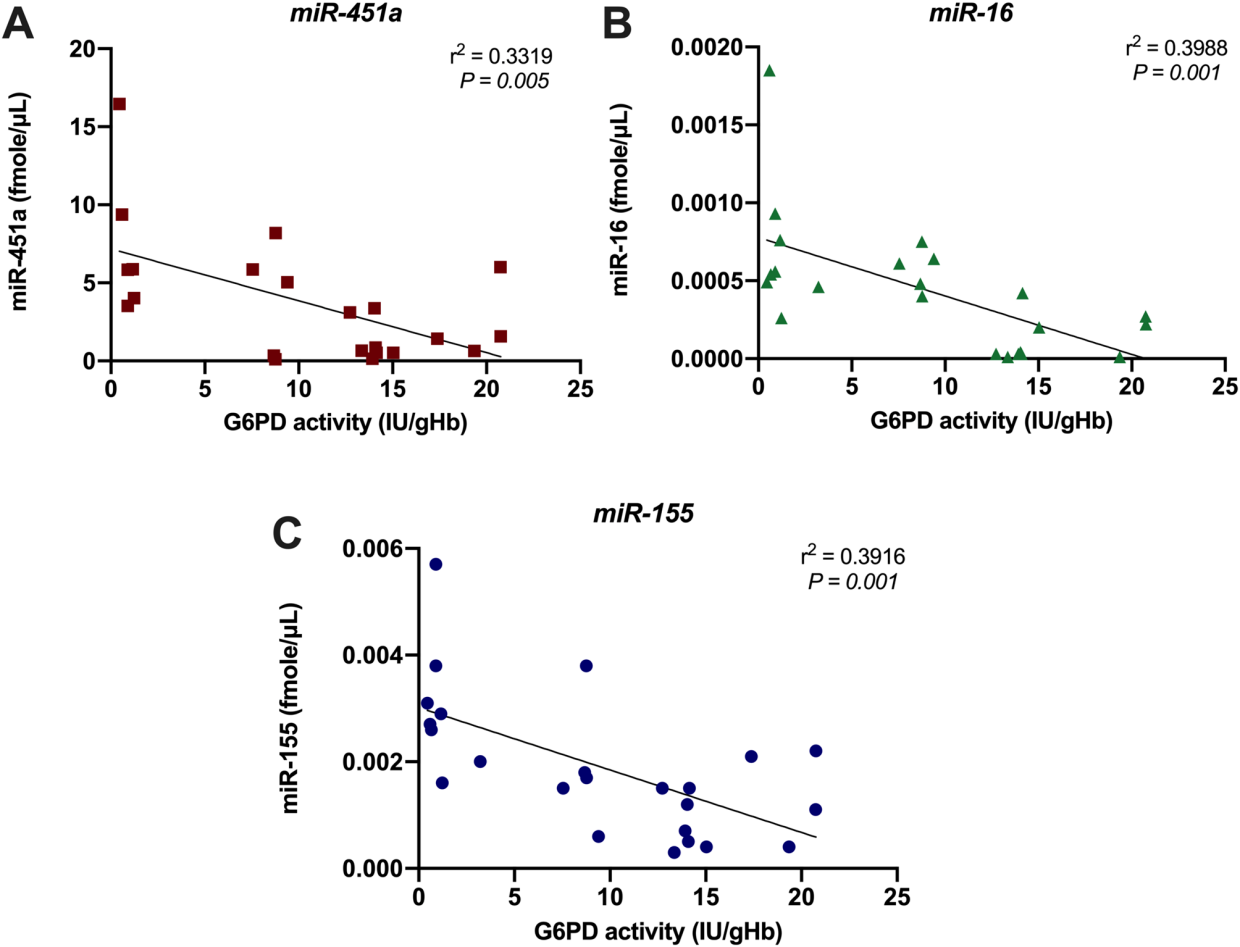
Correlations of quantitative G6PD activity with serum (**A**) miR-451a, (**B**) miR-16, and (**C**) miR-155 levels. Correlations between the levels of each miRNA and G6PD activity were determined using simple linear regression. A *P* value < 0.05 was considered to indicate statistical significance.

To evaluate the relationship between miRNA expression and G6PD activity, correlation coefficients for miR-451a expression were determined. There were significant associations of G6PD enzyme activity with miR-451a (r^2^ = 0.3319, *P* = 0.005) (Fig. 2A), miR-16 (r^2^ = 0.3988, *P* = 0.001) (Fig. 2B), and miR-155 (r^2^ = 0.3916, *P* = 0.001) levels (Fig. 2C).

### MiR-451a, miR-16, and miR-155 as promising biomarkers

**Figure 3:**
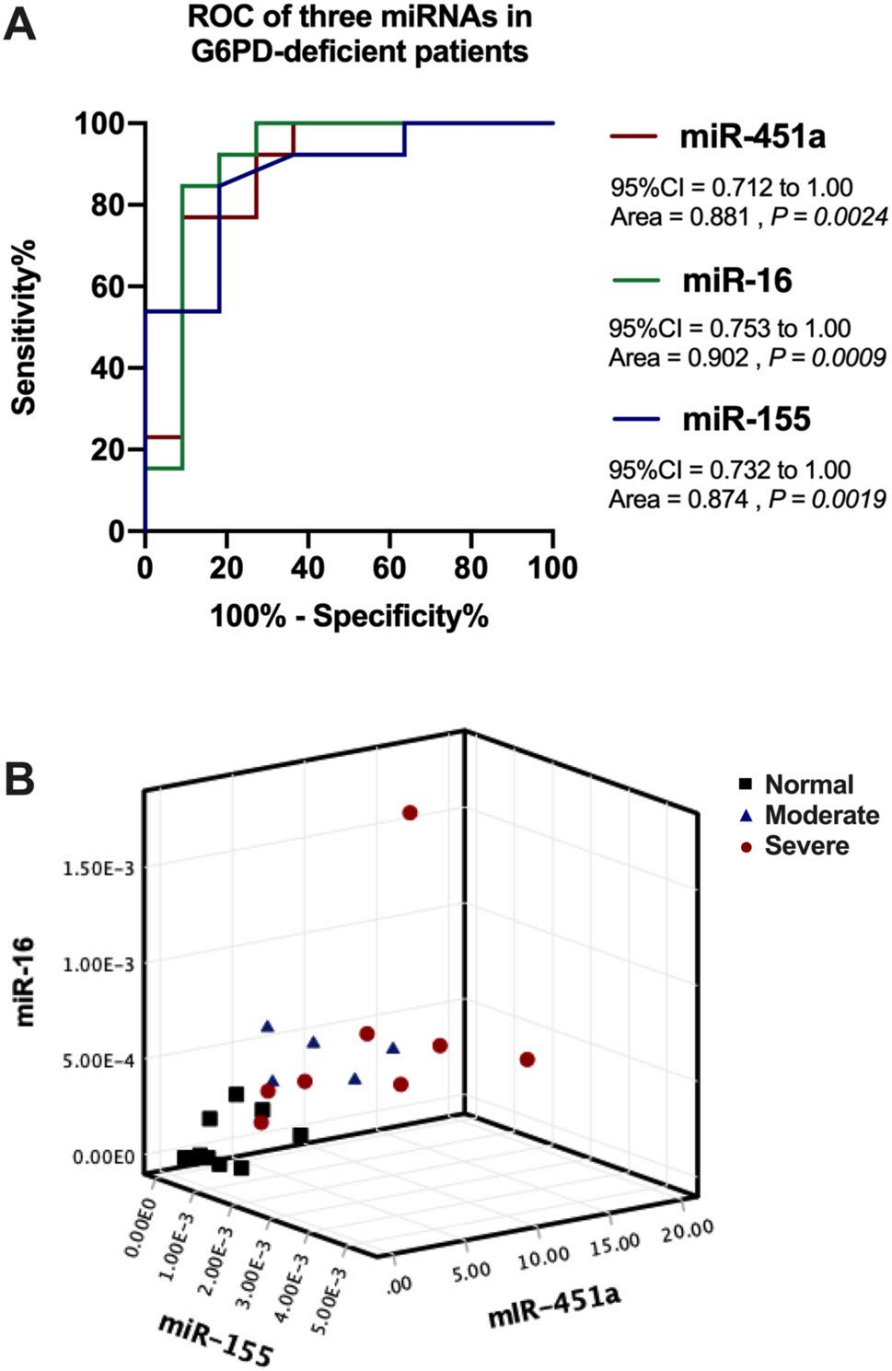
A set of three miRNAs (miR-451a, miR-16, and miR-155) distinguishes G6PD-deficient individuals from normal controls. (**A**) ROC curve analysis of miR-451a, miR-16, and miR-155 levels. The sensitivity and specificity were calculated to determine the AUC with 95% confidence intervals (CIs). A *P* value < 0.05 was considered to indicate statistical significance. (**B**) A 3D scatter plot of the three miRNAs was generated using SPSS version 22 to identify patients with G6PD deficiency.

To evaluate the prognostic value of serum miR-451, miR-16, or miR-155, the delta Ct values of normal and G6PD-deficient subjects were used to generate receiver operating characteristic (ROC) curves. The miR-16 data separated G6PD-deficient patients from normal individuals with a high sensitivity and an area under the curve (AUC) of 0.902 (*P* = 0.0009) (Fig. 3A). The miR-451a and miR-155 levels distinguished the normal and G6PD-deficient groups with AUC values of 0.881 (*P* = 0.0024) and 0.874 (*P* = 0.0019), respectively. Among the three miRNAs, miR-16 exhibited the highest sensitivity in detecting G6PD deficiency. We determined the optimal cut-off value for these three miRNAs based on high sensitivity and specificity, as well as Youden's index^26^, as indicated in Supplementary Information 2. The cut-off values for distinguishing patients with G6PD deficiency using miR-451a (2.73 fmol/µL), miR-16 (0.00041 fmol/µL), and miR-155 (0.00155 fmol/µL) are shown in Table 3. Using all three miRNAs as a set separated the G6PD-deficient group from the normal group (Fig. 3B, black squares), but this set of three miRNAs did not distinguish moderate from severe disease. These findings suggested that the panel of three miRNAs (miR-451a, miR-16, and miR-155) has the potential to distinguish G6PD-deficient patients, particularly those with severe G6PD deficiency.

**Table 3:** Diagnostic parameters for evaluating the ability of three miRNAs to distinguish G6PD deficiency.

miRNAs	AUC	*P* value	95% CI	Youden’s index	Cut-off	Sensitivity (%)	Specificity (%)
miR-451a	0.881	0.0024	0.712–1.00	3.2	2.73	76.9	72.7
miR-16	0.902	0.0009	0.753–1.00	1.8	0.00041	84.6	81.8
miR-155	0.874	0.0019	0.732–1.00	1.8	0.00155	84.6	81.8

*miRNA* microRNA, *AUC* area under the curve, *CI* confidence interval

## Discussion

In Thailand, the prevalence of G6PD deficiency ranges from 3 to 18%^2^ in adults to 10.7–17.0% in newborns^3,27,28^. The G6PD *Viangchan* (871G > A) variant is the most prevalent variant in the Thai population and is associated with severe disease^3,29^. Most G6PD-deficient adults are asymptomatic in their normal state and are unaware of this hereditary disease throughout their life^4^. Approximately 65% of G6PD-deficient Thai newborns exhibit severe neonatal jaundice, and 21.2–22% develop hyperbilirubinemia^3,30^. Therefore, screening for G6PD deficiency in newborns with severe hyperbilirubinemia is important. G6PD is the key enzyme for regulating glutathione sulfhydryl (GSH) levels in erythrocytes. Low G6PD activity increases the sensitivity of mature erythrocytes to oxidative stress, resulting in hemolysis in vivo, and an in vitro erythroid culture system has shown that G6PD is dispensable for the production of erythrocytes from erythroid progenitors^31^. In adulthood, individuals with G6PD deficiency do not have any hematological parameters^32,33^. The present study demonstrated that all complete blood count (CBC) data, including hemoglobin (Hb) levels and other red blood cell parameters, were within the normal range, consistent with the findings of previous studies^29,33^. Often with no obvious signs of hemolysis, G6PD-deficient patients are particularly vulnerable to hemolysis when exposed to oxidative agents.

Hemoglobin (Hb) is the major erythrocyte protein that accounts for nearly one-third of the weight of an erythrocyte^34^. The level of free Hb is a useful clinical biomarker of intravascular hemolysis, whereas the serum unconjugated bilirubin concentration is useful for estimating extravascular hemolysis^35^. The present study demonstrated that there was no difference in the hemolysis indices or free Hb levels in the nonacute phase of G6PD deficiency (Table 2), which were less than 5 mg/dL, and these results were consistent with the findings of previous studies^36^. However, the levels of miR-451a, miR-16, and miR-155 were elevated in G6PD-deficient patients (Fig. 1A). Because these miRNAs are abundant in mature erythrocytes^14,18^, the present findings suggested that the serum miR-451a, miR-16, and miR-155 levels increase due to subclinical hemolysis in G6PD-deficient patients even during the nonacute hemolytic phase. For other blood cell miRNAs, the present data showed that the levels of miR-144 (abundant in immature erythroid cells), miR-223 (derived from granulocytes), and miR-126 (derived from platelets) were not different from those in normal subjects. The altered expression of circulating miRNAs has been linked to the age of subjects^37^. We aimed to mitigate potential confounding factors, such as age. Our results demonstrated that age was not correlated with the levels of miRNAs in G6PD-deficient patients, as depicted in Supplementary Fig. 1. Additionally, the function of miRNAs released from circulating RBCs remains unclear. One possible function of miR-451a in RBCs is the suppression of oxidization because one of the target genes of miR-451a, namely, FOXO3, is related to the antioxidative pathway^23^. Several reports have shown that miR-451a binds to the Ago2 protein and is protected from the catalytic activity of RNase^38,39^. Circulating miR-451a may act as an antioxidant molecule if it is transferred to cells in distant organs as well as to RBCs.

Previous studies have demonstrated that microRNAs are linked to pathophysiology, and several studies have characterized disease-specific abnormalities in plasma miRNAs^40,41^. In various hemolytic anemias, including thalassemia, sickle cell anemia, and paroxysmal nocturnal hemoglobinuria (PNH), abnormal miRNA profiles have been investigated. For example, elevated levels of circulating miR-451a are associated with severe types of beta-thalassemia^11,12^. A previous study has shown that low levels of miR-510 and miR-629 are associated with a greater risk of severe sickle cell disease ^10^. MiR-148b-3p and miR-126-3p are more differentially expressed in PNH patients than in control subjects^13^. In patients with normal erythropoiesis, miR-155 and miR-451 play crucial roles in erythroid differentiation. miR-451 is upregulated in a lineage-specific manner during erythroid maturation, while miR-155 is downregulated during the early stages of erythropoiesis^18,24^. Due to ineffective erythropoiesis in thalassemia, significantly higher levels of plasma miR-451 and miR-155 are observed in β0-thalassemia/HbE patients^11^. Elevated plasma miR-451 levels may originate from the destruction of erythroid cells, while increased serum miR-155 levels can be attributed to the high expression of miR-155 in proliferating nucleated erythroblasts^11^. Concerning G6PD deficiency, our previous study confirmed no signs of ineffective erythropoiesis. The CD34-positive hematopoietic stem and progenitor cells from patients with G6PD deficiency could differentiate into mature erythrocytes in vitro^31^. Although severe G6PD-deficient patients did not exhibit increased hemolysis or ineffective erythropoiesis in the nonacute phase, the present findings of severe G6PD deficiency revealed increased levels of miR-451a (approximately 6 fmol/µL). Takada et al. (2021) reported that miR-451a levels are increased in individuals with other hemolytic disorders, such as autoimmune hemolytic anemia (AIHA) (30 fmol/µL), PNH (320 fmol/µL), α-thalassemia (280 fmol/µL), β-thalassemia (150 fmol/µL), and malaria (60 fmol/µL)^12^. The levels of miR-451a in patients with severe G6PD deficiency are lower than those reported in other hemolytic disorders characterized by intravascular hemolysis, such as AIHA, PNH, malaria infection, and the ineffective erythropoiesis observed in β-thalassemia. Further analysis including acute-phase patients is necessary to confirm the clinical relevance of the analysis of these miRNAs in patients with G6PD deficiency.

Drug-induced hemolysis caused by G6PD deficiency often occurs between 24 and 72 h after exposure to antimalarial drugs^42^, and G6PD levels may normalize during an acute hemolytic episode due to the increased G6PD activity in reticulocytes compared to that in mature red blood cells. Using serum miRNAs during the crisis phase of the drug-induced hemolytic phase may be helpful in the future to identify which patients have G6PD deficiency. Additionally, the heterozygous genotype is generated by inheritance, whereas the phenotype is defined by the pattern of X chromosome inactivation. Knowing heterozygosity in a female's genotype does not provide a reliable prediction of whether she will experience severe hemolysis with primaquine^43^. However, if a patient tends to have G6PD deficiency, the circulating miRNA concentration can be used as a predictive factor.

Screening for G6PD deficiency is not necessarily performed for all newborns in Thailand. The WHO has recommended routine screening for G6PD deficiency in infants residing in areas where the prevalence of the condition is as high as 3–5% in males to prevent adverse effects^44^. The fluorescent spot test and the enzyme activity assay are effective at detecting G6PD deficiency in newborns^45,46^. Although these technologies are sufficiently accurate and sensitive for screening, a multistep protocol that includes an erythrocyte hemolysis step is required. Circulating miRNAs have distinct advantages as potential clinical biomarkers because they are sensitive and accurate, and they can be measured with minimal blood volume. In Thailand, the diagnosis of G6PD deficiency typically occurs in symptomatic patients, often coinciding with neonatal jaundice. Nationwide neonatal screening for G6PD deficiency has not been implemented^2^. Consequently, there are avoidable hospital costs associated with phototherapy and hospitalization when infants are subsequently determined to have normal G6PD status. In particular, it would be beneficial to employ this panel of three miRNAs for distinguishing severe G6PD-deficient patients from healthy individuals, as shown in Fig. 3A,B. Therefore, the utilization of miRNA analysis may contribute to the development of an efficient, cost-effective technology that minimizes blood volume for identifying newborns with G6PD deficiency in the future.

However, hemolysis frequently occurs at the preanalytical step of blood sample processing and influences the levels of certain miRNAs detectable in serum and plasma^9,25^. Takada et al. demonstrated that miR-451a expression increases 1.62-fold before serum separation for 1 h after blood draws^12^. The present data showed that the fold increase in miR-451a expression was significantly greater than that in normal controls (10.65 and 12.21 for moderate and severe G6PD deficiency, respectively) (data not shown), which indicated that the increase in miR-451a expression was not a result of sample processing. Thus, the elevated levels of serum miR-451a in hemolytic disease patients were sufficiently high to distinguish pathological hemolysis from preanalytical variation. To standardize the use of miRNAs for detecting subclinical hemolysis, studies with larger cohorts and longitudinal studies of G6PD-deficient patients, including those in stable and acute hemolytic phases, are needed. Moreover, the utility of this set of miRNAs for disease specificity and their applicability to other hemolytic conditions should be confirmed through further in vitro and animal model studies.

In conclusion, the present study demonstrated for the first time that a set of three miRNAs (miR-451a, miR-16, and miR-155) is useful for identifying subclinical hemolysis in G6PD-deficient subjects. Overall, miRNA-based biomarkers show promise as tools for identifying individuals in the non-acute phase of G6PD deficiency. These miRNAs could aid in translational medicine for patients experiencing hemolytic crises by integrating miRNA data with family history, hematological indices, and changes in hemoglobin levels. Additionally, determining G6PD status through miRNA analysis would be advantageous by enabling patients to avoid oxidative exposure. In addition, large cohort studies of miRNAs may help discriminate other hemolytic diseases, such as pyruvate kinase deficiency, hereditary spherocytosis, or elliptocytosis.

## Methods

### Subjects

The present study enrolled 13 patients with the G6PD *Viangchan* (871G > A) variant (hemizygote = 7, heterozygote = 5, homozygote = 1) and 11 subjects with normal G6PD. Eight of the thirteen subjects with the *Viangchan* variant had severe G6PD deficiency, and five of these subjects had moderate G6PD deficiency. The genetic data of all the subjects were obtained in a previous study^47^. Patients with hemoglobinopathies and other noncommunicable chronic diseases were excluded from the present study^47^. None of the enrolled subjects had been hospitalized for more than a month or had concomitant infections. All procedures involving human subjects were performed in accordance with the ethical standards of the Helsinki Declaration of 1975. The research protocol was approved by the Ethical Review Committees for research involving human subjects at Chulalongkorn University (COA no. 200/65 and COA no. 196/66) and the International University of Health and Welfare (22-Ifh-050). Prior to participation, all the subjects provided informed consent after receiving information on the purpose, potential risks, and benefits of the study.

### Measurement of G6PD activity

EDTA blood samples were collected for measurements of G6PD activity, which determined the kinetic change in NADP + to NADPH within 10 min^48^. NADPH was detected at a wavelength of 340 nm using a Thermo Evolution 600 UV‒Vis Spectrophotometer. G6PD activity is expressed as international units per gram of hemoglobin (IU/g Hb). According to World Health Organization (WHO) guidelines^49^, G6PD deficiency was defined as G6PD activity less than 1.5 IU/g Hb.

### Serum preparation for miRNA analyses

Serum obtained from clotted whole blood was used for miRNA analyses^12^. Briefly, 12 mL of whole blood was drawn using a vacuum sample tube containing coagulation stimulators. The serum was separated from the primary tube within 1 h by centrifugation at 3,500 × g for 10 min. To remove cell debris, the serum was centrifuged at 10,000 × g for 5 min at 4 °C, and the cleared supernatant was transferred to a new tube prior to miRNA analyses.

### Measurement of hemolysis indices

Serum (600 µL) was used for measurements of hemolysis indices, including aspartate aminotransferase (AST), lactate dehydrogenase (LDH), and potassium (K +) levels. The serum free Hb concentration was measured at an optic density of 414 nm by using a NanoDrop apparatus^12,50^ (ND2000c, Thermo Fisher Scientific Co., Waltham, MA, USA).

### MiRNA analyses

miRNA levels were determined using reverse transcriptase-based quantitative polymerase chain reaction (RT‒qPCR)^11,12^. Briefly, serum miRNAs were extracted with a Nucleospin™ plasma extraction kit (Macherey–Nagel, Takara, Shiga, Japan). One femtomole of cel-miR-39 (CosmoBio, Tokyo, Japan) was added as the spiked-in control during the extraction process. Purified miRNAs were converted into complementary DNAs using a TaqMan reverse transcriptase kit (Thermo Fisher Scientific Co., Waltham, MA, USA). qPCR analysis was performed using TaqMan microRNA assay kits and Universal PCR Master Mix (Thermo Fisher Scientific, Waltham, MA, USA). After the initial denaturation step at 95 °C for 10 min, 40 PCR cycles were performed at 95 °C for 15 s and 60 °C for 60 s, using a real-time PCR machine (ABI7500fast, Thermo Fisher Scientific Co., Waltham, MA, USA). Each miRNA measurement was performed in triplicate. As a negative control, nuclease-free water was used. The expression of each miRNA was compared to the expression of the spiked in cel-miR-39 using the comparative Ct method^11^. The absolute levels of each miRNA were calculated using a concentration of 0.5 fmol/μL cel-miR-39.

### Statistical analyses

The normality of the data was tested using the Kolmogorov‒Smirnov test. Kruskal‒Wallis tests were used to evaluate differences among patients with normal G6PD, moderate G6PD deficiency, and severe G6PD deficiency. Receiver operating characteristic (ROC) curves were constructed to evaluate the diagnostic accuracy of each miRNA. Differences were considered significant at *P* < 0.05. The data were visualized using Prism software (version 8.0; GraphPad Software, Inc., CA, USA).

### Supplementary Information

Supplementary Figure 1.

Supplementary Information 2.

## Data Availability

The data presented in this study are available upon request from the corresponding author.
