# Supplementary figures and images for: Serum microRNAs as new biomarkers for detecting subclinical hemolysis in the nonacute phase of G6PD deficiency
Source: Sci Rep. 2024 Jul 11;14:16029. doi: 10.1038/s41598-024-67108-4 (PMC11239928; doi:10.1038/s41598-024-67108-4)

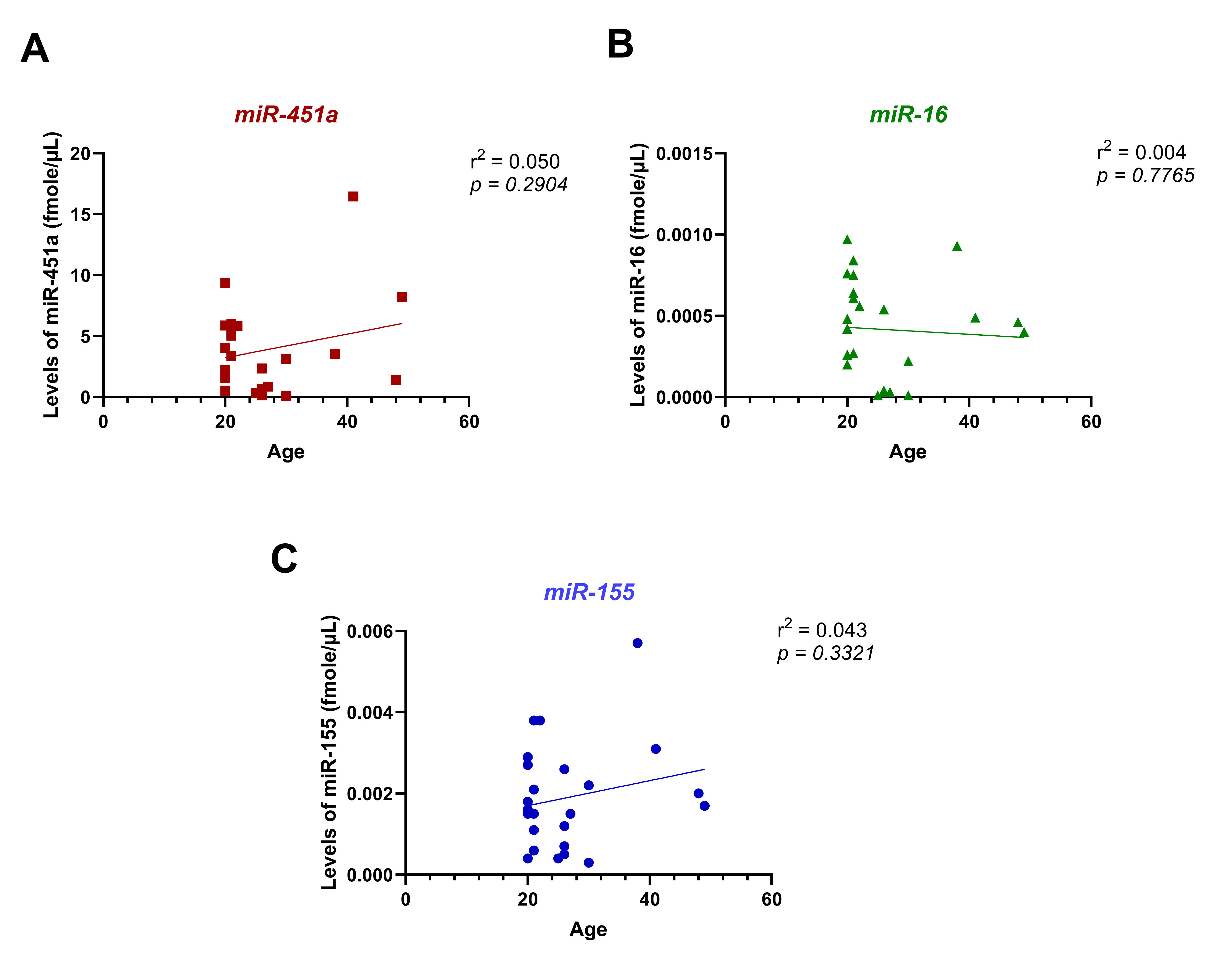

Supplement: Supplementary file 1 — Supplementary Figure 1. [file 41598_2024_67108_MOESM1_ESM.tif]
